# Das Verfahren geht weit über „die App“ hinaus – Datenschutzfragen von Corona-Tracing-Apps: Einführung in Datenschutz-Folgenabschätzungen als Mittel, gesellschaftliche Implikationen zu diskutieren

**DOI:** 10.1007/s00287-020-01313-z

**Published:** 2020-10-12

**Authors:** Kirsten Bock, Christian Ricardo Kühne, Rainer Mühlhoff, Měto R. Ost, Jörg Pohle, Rainer Rehak

**Affiliations:** 1 Weizenbaum-Institut für die vernetzte Gesellschaft, Berlin, Deutschland; 2 Wissenschaftszentrum Berlin für Sozialforschung gGmbH, Berlin, Deutschland

## Abstract

Seit der Ausbreitung des SARS-CoV-2-Virus in Europa Anfang 2020 wir dan technischen Lösungen zur Eindämmung der Pandemie gearbeitet. Unter den verschiedenen Systementwürfen stechen jene hervor, die damit werben, datenschutzfreundlich und DSGVO-konform zu sein. Die DSGVO selbst verpflichtet die Betreiberïnnen umfangreicher Datenverarbeitungssysteme wie etwa Tracing-Apps zur Anfertigung einer Datenschutz-Folgenabschätzung (DSFA) aufgrund des hohen Risikos für die Rechte- und Freiheiten (Art. 35 DSGVO). Hierbei handelt es sich um eine strukturierte Risikoanalyse, die mögliche grundrechtsrelevante Folgen einer Datenverarbeitung im Vorfeld identifiziert und bewertet.

Wir zeigen in unserer DSFA, dass auch die aktuelle, dezentrale Implementierung der Corona-Warn-App zahlreiche gravierende Schwachstellen und Risiken birgt. Auf der rechtlichen Seite haben wir die Legitimationsgrundlage einer freiwilligen Einwilligung untersucht und formulieren die begründete Forderung, dass der Einsatz einer Tracing-App gesetzlich geregelt werden muss. Weiterhin wurden Maßnahmen zur Verwirklichung von Betroffenenrechten nicht ausreichend betrachtet. Nicht zuletzt ist die Behauptung, ein Datum sei anonym, hoch voraussetzungsreich. Anonymisierung muss als ein kontinuierlicher Vorgang begriffen werden, der eine Abtrennung des Personenbezugs zum Ziel hat und auf dem Zusammenspiel von rechtlichen, organisatorischen und technischen Maßnahmen beruht. Der derzeit vorliegenden Corona-Warn-App fehlt es an einem solchen expliziten Trennungsvorgang. Unsere DSFA zeigt dabei auch die wesentlichen Defizite der offiziellen DSFA der Corona-Warn-App auf.

## Einleitung

Seit einigen Monaten kreist die Diskussion über die Eindämmung der Corona-Pandemie um den Einsatz technischer Hilfsmittel, insbesondere von sogenannten Corona-Tracing-Apps. Diese sollen automatisiert die epidemiologisch relevanten Kontaktereignisse von Nutzer*innen aufzeichnen und es so erlauben, im Infektionsfall Einzelner zeitnah und rückwirkend die exponierten Kontaktpersonen warnen und isolieren zu können. Bislang wurde das sogenannte Contact-Tracing allein manuell von Mitarbeiter*innen der Gesundheitsbehörden vollzogen, also etwa anhand des Gedächtnisses der Infizierten und anschließender Warnung per Telefon. In einigen Ländern, etwa China, werden auch viele weitere Informationsquellen und Datenkategorien wie beispielsweise Kreditkartendaten oder Reiseinformationen genutzt. Diese mühsame Arbeit kann, so die Vision, durch den Einsatz von Apps wesentlich beschleunigt werden.

Auch wenn die konkrete Tauglichkeit einer solchen App für diesen Zweck sowohl epidemiologisch als auch technisch noch umstritten ist und die Gefahr einer grundsätzlichen gesellschaftlichen Gewöhnung an Contact-Tracing besteht, soll es an dieser Stelle nicht um ein generelles „Ob“, sondern ein „Wie“ einer solchen App gehen. Denn erst bei der Betrachtung der konkreten technischen Umsetzung lassen sich individuelle und gesellschaftliche Konsequenzen analysieren. Die Erkenntnisse können dann wiederum in Form von Anforderungen zurück in die konkrete Ausgestaltung und Weiterentwicklung des gesamten Verfahrens fließen.

Datenschutz und seine Verankerung in der Gesetzgebung ist ein Garant der Grundrechte und Grundfreiheiten im digitalen Zeitalter. Er bezieht sich nicht nur auf individuelle, sondern auch auf kollektive Rechte. Datenschutz hält auch die funktionelle Differenzierung moderner Gesellschaften aufrecht, indem er strukturelle Machtasymmetrien problematisiert und somit gesellschaftliche Grundfunktionen absichert [4]. Im Unterschied zu Fragen der IT-Sicherheit, die die verarbeitende Organisation schützt, geht es dem Datenschutz weniger um externe Angriffe auf Systeme und Daten, sondern primär um Grundrechtseinschränkungen bezüglich der betroffenen Personen durch die Datenverarbeitung selbst. Im Fokus steht deshalb nicht primär die „Privatheit“ des einzelnen Datensubjekts, sondern die gesamtgesellschaftlichen, strukturellen Auswirkungen und Machteffekte einer Datenverarbeitung [7]. Eine Datenschutzanalyse geht somit prinzipiell von der verarbeitenden Organisation als der primären Risikoquelle aus, um den Blick von dort schließlich auch auf Plattformen, Dienstleister*innen, Nutzer*innen und externe Dritte zu richten [5].

## Datenschutz-Folgenabschätzung

Auch wenn viele Eigenschaften der Tracing-Apps, darunter ihre genaue Zweckbestimmung, mittlerweile feststehen, steht eine angemessene Analyse der datenschutz- und somit grundrechtsrelevanten Folgen dieses Vorhabens nach wie vor aus. Die offizielle Datenschutz-Folgenabschätzung (DSFA), die das Robert Koch-Institut (RKI) als verantwortliche Stelle kurz vor der Veröffentlichung der App vorlegte, ist aufgrund von wesentlichen methodischen, technischen und rechtlichen Defiziten dafür leider ungeeignet [2]. Dabei müssen die diversen Folgen schon vor einem breiten Einsatz detailliert diskutiert werden, insbesondere bei diesem nationalen bis europäischen Projekt zur großflächigen Kontaktnachverfolgung unter staatlicher Verantwortung. Für diese Art der Analyse gibt es in der europäischen Datenschutzgrundverordnung (DSGVO) das Instrument der Datenschutz-Folgenabschätzung. Dort heißt es in Art. 35 DSGVO („Datenschutz-Folgenabschätzung“):

(1) Hat eine Form der Verarbeitung, insbesondere bei Verwendung neuer Technologien, aufgrund der Art, des Umfangs, der Umstände und der Zwecke der Verarbeitung voraussichtlich ein hohes Risiko für die Rechte und Freiheiten natürlicher Personen zur Folge, so führt der Verantwortliche vorab eine Abschätzung der Folgen der vorgesehenen Verarbeitungsvorgänge für den Schutz personenbezogener Daten durch.

Für das methodische Vorgehen im Rahmen einer DSFA gibt es unterschiedliche Ansätze. In Deutschland wird dafür von der Konferenz der Datenschutzbeauftragten des Bundes und der Länder das von ihr ausgearbeitete „Standard-Datenschutzmodell“ (SDM) empfohlen, an dem auch wir uns im Folgenden orientieren. Dieses Modell verlangt zunächst eine Schwellenwertanalyse, um zu klären, inwiefern eine DSFA für ein gegebenes Datenverarbeitungssystem nicht nur gesellschaftlich wünschenswert, sondern auch datenschutzrechtlich gefordert ist. Weil mit den Contact-Tracing-Apps sowohl eine neuartige Technologie sowie personenbezogene Daten in großem Umfang und im Infektionsfall sogar medizinische Daten verarbeitet werden, ist dies hier unzweifelhaft der Fall. Trotzdem hatte noch bis zum April dieses Jahres während der grundsätzlichen Architekturdiskussion keine verantwortliche Stelle eine DSFA für eine der in Deutschland diskutierten Apps vorgelegt. Um die gebotene Aufmerksamkeit auf dieses Thema zu legen, haben wir, eine Gruppe von Wissenschaftler*innen und Datenschützer*innen, dann im April kurzerhand selbst eine Muster-DSFA zur Corona-App erarbeitet und in die öffentliche Diskussion eingebracht [1].

Die Struktur der vorgelegten DSFA folgt den Vorgaben des Kurzpapiers Nr. 5 der Konferenz der Datenschutzbeauftragten des Bundes und der Länder, das Risikokalkül und die Bestimmung der Schutzmaßnahmen folgen dem Standard-Datenschutzmodell (SDM) [3].

Im ersten Schritt wird der Zweck des gesamten Datenverarbeitungsverfahrens definiert, in diesem Falle ausschließlich das Erkennen und Unterbrechen von Infektionsketten. Danach gilt es, den Kontext der Verarbeitung herauszuarbeiten. Dies umfasst nicht nur die allgemeine gesellschaftliche und politische Lage sowie technische Umstände, sondern auch explizit die verschiedenen Akteure und ihre Interessen. Erst auf dieser Grundlage kann später eine fundierte Analyse von Risiken und Angriffsszenarien erstellt werden.

Sodann müssen Annahmen und Anwendungsfälle für die Verarbeitung erarbeitet werden, um daran anschließend die Verarbeitungstätigkeit im Detail zu beschreiben. Dabei ist zu beachten, das Verfahren in Teilschritte zu zerlegen sind, von denen nicht alle technikgestützt ablaufen müssen. Im vorliegenden Fall umfasst das Verfahren nicht nur die App, sondern auch die dazugehörigen Serversysteme, Fachanwendungen und Infrastrukturbestandteile wie etwa Betriebssysteme oder technische Kommunikationsbeziehungen. Auf dieser Basis werden dann Rechtsgrundlagen und die Verantwortlichkeit der Verarbeitungstätigkeit diskutiert sowie rechtliche Anforderungen erarbeitet.

All diese Vorarbeiten kombinierend, werden Schwachstellen, Gefahren und Risiken der Verarbeitung entwickelt. Damit sind Risiken bezüglich der Grundrechte der Betroffenen gemeint, und zwar aller Grundrechte. Auf dre Risikoanalyse aufbauend werden dann Schutzmaßnahmen für die Rechte der Betroffenen bestimmt und zuletzt Empfehlungen für die Verantwortlichen aufgeführt. Die Empfehlungen umfassen insbesondere die besonders problematischen Aspekte, etwa Risiken, für die keine Schutzmaßnahmen existieren.

## Zentral oder dezentral?

Aus Gründen der Minimierung des Grundrechtseingriffs und zur Vereinfachung der Analyse gehen wir in unserer DSFA von einem eng umrissenen Zweck für die Datenverarbeitung aus: die Warnung von Personen, die mit Infizierten Kontakt hatten. Die Grundfunktionalität einer solchen App wird im Idealfall umgesetzt, indem das Smartphone in regelmäßigen Abständen über den „Bluetooth Low Energy Beacons“-Standard wechselnde Zeichenfolgen (temporäre Kennungen, tempIDs) via Bluetooth versendet und entsprechend die temporären Kennungen (tempIDs) von anderen Apps empfängt, sofern diese örtlich nah genug sind. Diese Daten ermöglichen eine Kontaktnachverfolgung; aus Dauer und Nähe des Kontakts sowie der Krankheitsphase der infizierten Person soll ein Ansteckungsrisiko berechnet werden. Ortsinformationen, also zum Beispiel der GPS-Standort, werden durch dieses System nicht erhoben. Die zugrunde liegende Bluetooth-Funktionalität wird dabei durch das von Apple und Google im jeweiligen mobilen Betriebssystem bereitgestellte Exposure Notification Framework (ENF) bereitgestellt.

An dieses grundlegende Prinzip zur Detektion von Kontaktereignissen mittels Bluetooth schließen sich nun technische Fragen an, in denen verschiedene Varianten diskutiert wurden. Im Mittelpunkt der Diskussion stand die Frage, ob die Berechnungen des individuellen Expositionsrisikos lokal auf den Mobiltelefonen der Nutzer*innen, oder serverseitig stattfindet. Damit hängt auch die Frage zusammen, wie genau die exponierten Nutzer*innen kontaktiert werden, um sie zu warnen.

In der zentralen Architektur werden im Fall der positiven Testung alle Kontaktereignisse von der App der infizierten Person auf einen Server hochgeladen. Dieser Server berechnet das Expositionsrisiko für alle Kontakte dieser Person und informiert diese dann aktiv. Der Server bzw. die Infrastruktur hat in dieser Variante somit Kenntnis der Infizierten, derer Kontakte und des sozialen Graphen.

Die dezentrale Architektur dagegen sieht vor, dass im Falle der positiven Testung nur die von der Person in den vergangenen 14 Tagen ausgesendeten temporären Kennungen (tempIDs) auf den Server geladen werden. Die anderen Apps laden sich regelmäßig einen Datensatz aller tempIDs von infizierten Nutzer*innen herunter und berechnen lokal auf ihrem Smartphone, ob ein Risiko der Ansteckung vorliegt. Der Server kennt in dieser Variante nur die temporären Kennungen der Infizierten, er kann weder ihre Kontakthistorie noch das Kontaktnetzwerk der Nutzer*innen nachvollziehen. Aus diesem Grunde ist die dezentrale Variante deutlich datenschutzfreundlicher (allerdings auch Datenverkehrsintensiver).

Unsere DSFA betrachtet nur den dezentralen, grundrechtsschonenderen Ansatz, der von vielen europäischen Ländern wie etwa Österreich, Schweiz, Estland und seit Ende April auch von Deutschland verfolgt wird.

## Zentrale Erkenntnisse

Im Folgenden sollen vier wichtige Ergebnisse unserer DSFA vorgestellt werden, die leider nach wie vor relevant und aktuell sind.

1. Die häufig beteuerte Freiwilligkeit der App-Nutzung ist ein voraussetzungsreiches Konstrukt, das sich in der Praxis als Illusion herausstellen kann. So ist vorstellbar und wird auch bereits diskutiert, dass die Nutzung der App als Bedingung für die individuelle Lockerung der Ausgangsbeschränkungen gelten könnte. Das Vorzeigen der App könnte als Zugangsbedingung für öffentliche oder private Gebäude, Räume oder Veranstaltungen dienen. Eine solche Verwendungsweise wäre mitunter nicht durch den Zweck des Systems gedeckt, könnte aber durch dritte Akteure (z. B. Arbeitgeber oder private Veranstalter) in Kraft gesetzt werden. Dieses Szenario würde eine implizite Nötigung zur Nutzung der App bedeuten und zu einer erheblichen Ungleichbehandlung der Nicht-Nutzer*innen führen; die ohnehin vorhandene „digitale Schere“ zwischen Smartphone-Besitzer*innen und -Nicht-Besitzer*innen würde sich hiermit auf weitere Lebensbereiche ausweiten. Zudem könnte der Zweck des Systems unterminiert werden, wenn Nutzer*innen aus Angst vor Nachteilen ihr Smartphone absichtlich nicht bei sich führten oder abwechselnd verschiedene Geräte nutzten. Nur durch eine flankierende Gesetzgebung, die diese und andere Zweckentfremdungen effektiv unterbindet, ist dieses Risiko abzumildern. Hierbei ist darauf hinzuweisen, dass die informierte Einwilligung *kein* geeigneter rechtlicher Rahmen für eine freiwillige App-Nutzung ist. Denn die informierte Einwilligung externalisiert das Risiko der (Grundrechts‑)Folgen sowie die Abwägung zwischen Nutzen und Folgen auf die Betroffenen. Dabei käme es darauf an, gerade diese Abwägung zum Gegenstand demokratischer Aushandlung zu machen. Als Rechtsgrundlage wäre deshalb ein Gesetz erforderlich, in dem die (demokratisch legitimierte und kontrollierte) Gesetzgeber*in die Verarbeitung festlegt und auch deren Grenzen definiert. Dies ist aktuell politisch jedoch nicht angedacht.
2. Ohne Intervenierbarkeit (Einschreitbarkeit) und enge Zweckbindung ist der Grundrechtsschutz gefährdet: Es besteht ein hohes Risiko, fälschlich registrierter Expositionsereignisse (falsch Positive durch Wände, Masken oder Laborfehler), die zu Unrecht auferlegte Selbstquarantäne zur Folge hätten. Um dem zu begegnen, bedarf es rechtlicher und faktischer Möglichkeiten zur effektiven Einflussnahme, etwa das Zurückrufen falscher Infektionsmeldungen, die Löschung falsch registrierter Kontaktereignisse oder das Anfechten möglicher anderer Konsequenzen. Keine bekannte App erlaubt dies bislang.
3. Alle bislang besprochenen Varianten einer Corona-App unterliegen der DSGVO, denn sie verarbeiten personenbezogene Daten. Alle Daten auf einem Smartphone sind personenbezogen, nämlich bezogen auf die Nutzer*in des Gerätes. Die gilt unabhängig davon, ob Beteiligte die versendeten Zeichenfolgen auf eine Person zurückführen können oder ob das Gerät gut vor Zugriff Dritter abgesichert ist. Und weil nur diejenigen Personen Daten an den Server übertragen, die als infiziert diagnostiziert wurden, handelt es sich bei diesen hochgeladenen Daten sogar um Gesundheitsdaten. Nur durch ein Zusammenspiel organisatorischer, rechtlicher und technischer Maßnahmen kann der Personenbezug wirksam und irreversibel von den hochgeladenen Daten abgetrennt werden, sodass sie letztlich auf dem Server nur noch als „infektions-indizierende Daten ohne Personenbezug“ ankommen. Dieses Anonymisierungsverfahren kann diverse Formen annehmen, muss jedoch kontinuierlich datenschutzrechtlich durch die zuständige Aufsichtsbehörden prüfbar sein: Organisatorisch müssen die Verantwortlichen in strategischer und die Betreiber*in(nen) in operativer Hinsicht eine Mischstruktur etablieren. Die Verantwortliche – etwa das RKI – könnte strategisch beispielsweise zwei unterschiedliche Betreiber*innen auswählen: Eine betreibt die Eingangsknoten im Netzwerk und trennt die Metadaten ab, darunter die IP-Adressen, die andere betreibt den eigentlichen Server. Auf der Ebene der Betreiber*innen muss dann operativ auf eine angemessene Abteilungsstruktur und Funktionstrennung geachtet werden, die die informationelle Gewaltenteilung innerhalb der Organisation – also die funktionale Differenzierung – durchsetzen [6]. Rechtlich müssen die Betreiber*innen unabhängig sein, keine eigenen Interessen an den Daten haben und vor Pflichten zur Herausgabe von Daten geschützt sein, auch gegenüber staatlichen Sicherheitsorganen. Technisch muss die Betreiber*in die Trennung so umsetzen, dass die Uploads nicht protokolliert werden können, weder auf dem Server noch in ihrem Netzwerk. All diese Maßnahmen müssen durch ein Datenschutzmanagementsystem kontinuierlich prüfbar gemacht werden und auch geprüft werden.
4. Die Rolle der Plattformanbieter Apple (iOS) und Google (Android) ist kritisch zu diskutieren und über den gesamten Verarbeitungsprozess hinweg zu begleiten. Eine Bluetooth-basierte Corona-Tracing-App ist aus technischen Gründen auf die Kooperation der Plattformanbieter angewiesen, da der Zugriff auf das Bluetooth-Modul der Geräte auf Betriebssystemebene ermöglicht werden muss. Diese Machtposition haben die Plattformanbieter in den vergangenen Wochen genutzt, um gegen zahlreiche Regierungen eine dezentrale und somit datenschutzfreundlichere Architektur zu erzwingen. Das ist im Ergebnis wünschenswert, aber im Prozess hochproblematisch. Zudem ist durch diesen Schachzug das Datenschutzrisiko, das von den Plattformbetreibern selbst ausgeht, in der öffentlichen Diskussion weitestgehend aus dem Blick geraten. Als Betriebssystemhersteller ist es prinzipiell möglich (und auch realistisch, wie die DSFA zeigt), dass Google und Apple an die Kontaktinformationen gelangen und daraus Informationen über Infektionsfälle und Expositionsrisiken ableiten können. Zudem wird der Quellcode des ENF nach wie vor unter Verschluss gehalten. Eine kritische Begleitung der Rolle von Apple und Google erfordert daher eine umfassende Sensibilisierung für dieses Problem und die rechtliche Verpflichtung der Unternehmen, sich datenschutzkonform zu verhalten.

## Abschluss

Insbesondere die quelloffene Entwicklung von Servern und Apps nebst allen ihren Komponenten – beispielsweise als freie Software – ist eine wesentliche Voraussetzung dafür, dass nicht nur für Datenschutzaufsichtsbehörden die nötige Transparenz bezüglich der Umsetzung der Datenschutzgrundsätze vorliegt, sondern auch für die Betroffenen und die Öffentlichkeit insgesamt. Diese Datenschutz-Folgenabschätzung zeigt aber auch, dass eine technische Fokussierung allein auf die Quelloffenheit der Software die durchaus größeren gesellschaftlichen Implikationen des gesamten Verfahrens verschleiern kann. Nur qualitativ hochwertige Datenschutz-Folgenabschätzungen können derartiges offenlegen und sollten in diesem, aber auch in anderen, ähnlich folgenreichen Datenverarbeitungsprojekten nicht nur angefertigt, sondern auch veröffentlicht werden, damit sie nicht nur von den Datenschutz-Aufsichtsbehörden, sondern auch in gesellschaftlicher Breite und Tiefe diskutiert werden können.
